# Knowledge about diabetic retinopathy, eye check-up practice and associated factors among adult patients with diabetes mellitus attending at debark hospital, Northwest Ethiopia

**DOI:** 10.1186/s12886-020-01730-4

**Published:** 2020-11-18

**Authors:** Abel Sinshaw Assem, Mebratu Mulusew Tegegne, Destaye Shiferaw Alemu, Asamere Tsegaw Woredekal, Tsehay Kassa Tefera

**Affiliations:** 1 Felege Hiwot Comprehensive Specialized Hospital, Bahirdar City, Ethiopia; 2 Department of Optometry, School of Medicine, College of Medicine & Health Sciences and Comprehensive Specialized Hospital, University of Gondar, Gondar, Ethiopia; 3 Department of Ophthalmology, School of Medicine, College of Medicine & Health Sciences and Comprehensive Specialized Hospital, University of Gondar, Gondar, Ethiopia

**Keywords:** Diabetic retinopathy, Knowledge, Practice, Debark, Ethiopia

## Abstract

**Background:**

Routine eye examination plays a vital role in detecting diabetic retinopathy in its earliest stage before the onset of blindness. Patients’ knowledge about the nature and the consequences of diabetic retinopathy and routine eye checkup helps for timely identification and early treatment. However, there is limited evidence on knowledge of patients with diabetes mellitus on diabetic retinopathy and their eye check-up practices in Ethiopia.

The aim of this study was to assess knowledge about diabetic retinopathy, eye check-up practice and associated factors of diabetic retinopathy among adult diabetic patients at Debark hospital, Northwest Ethiopia.

**Methods:**

Institution based cross-sectional study was conducted at Debark hospital, Northwest Ethiopia, from April 20/2018- May 20/2018. A pretested interviewer administered structured questionnaire was used to collect data among 230 diabetic patients aged 18 years and above. Data were entered in to Epi Info version 7 and exported to SPSS version 20 for analysis. Bivariable and multivariable binary logistic regression analyses were done. Odds ratio with 95% confidence level was determined and variables with *p*–value of < 0.05 were considered as statistically significant.

**Result:**

Out of 238 sample 230 were participated, among this, 119 (51.7%) were males. The mean age of the respondents was 49 (SD ±17.6) years. One hundred nine (47.4%) participants had good knowledge and 91 (39.6%) had good eye check-up practice. Urban residence [AOR = 2.65;95% CI: 1.16–6.07)]), monthly income of 3501–8000 birr [AOR = 4.54;(1.31–15.7)], type II diabetes mellitus [AOR = 3.9;(1.6–9.6)], duration of diabetes (6–12 years [AOR = 4.4;(1.4–13.5)]), history of eye disease [AOR = 5.5;(2.3–13.0)] were associated with good knowledge. Similarly, longer duration of diabetes (13–25 years [AOR = 3.77; (1.05–13.5)]) and history of eye disease [AOR = 2.47; (1.09–5.62)] were associated with good eye check-up practice.

**Conclusion:**

The proportion of good knowledge about diabetic retinopathy among diabetic patients at Debark hospital was fair (47.4%) and good eye check-up practice (eye examination at least once in the past year) was low (39.6%). Longer duration of diabetes and history of eye disease were identified as positive factors for good knowledge and eye check-up practice. Knowledge and regular eye check-up practice needs to be enhanced through provision of appropriate health education.

**Supplementary Information:**

The online version contains supplementary material available at 10.1186/s12886-020-01730-4.

## Background

Diabetic retinopathy (DR) is defined as a damage to the micro vascular system of the retina, accompanied by structural changes in the retina due to a prolonged hyperglycemia [1, 2]. Worldwide, 34.6%(93 million) of diabetic patients are living with DR [3]. In 2007, Out of 39 million global blindness due to various eye diseases, 4.8% was due to DR [4].

Nowadays**,** diabetes mellitus (DM) [5] and DR [6] are becoming a public health concern in developing countries. Estimates of proportion of African patients with diabetes who are visually impaired are high even compared with older European Americans [7]. Loss of productivity and quality of life due to DR leads to socioeconomic burdens on the community.

Routine eye examination is necessary for early detection of DR and prevention of blindness. This requires knowledge of sight threatening potential of DR and the need for regular eye examination. The knowledge and practice of regular eye cheek-up was reported as poor in developed countries [8]. This is expected to be worse in developing countries where most DM patients do not apply the recommended ocular examination aimed at preventing visual impairment and blindness from DR [9].

There are different factors which affects the knowledge and eye check-up practice of DR among diabetic patients [10, 11]. Sex being female [9, 12], longer duration of diabetes [13, 14] higher educational level [11, 13] and higher socioeconomic status [11, 15] were positively associated factors with good knowledge of DR. Older age [10, 11], language, ethnicity and residence being urban [16] were positively associated with good eye checkup practice.

Determining the level of knowledge, eye checkup practice and associated factors of DR will help as an input when the health authorities plan the prevention and elimination strategies of modifiable risk factors for poor knowledge and poor eye checkup practice. However, there is limited data regarding knowledge and eye cheek-up practice of DR among DM patients in Ethiopia. Therefore, this study was aimed to assess the knowledge, eye check-up practice and associated factors of diabetic retinopathy among adult diabetes mellitus patients attending diabetic follow-up clinic in Debark hospital.

## Methods

### Study design, setting and sampling

A hospital based cross sectional study was conducted to assess the knowledge, eye check-up practice and associated factors towards diabetic retinopathy among diabetic patients attending Debark hospital from April 20 to May 20, 2018, northwest Ethiopia.

A total of 238 sample size was determined using Open Epi computer software with a single population proportion formula considering a total population of adult diabetic patients 485, which is the total number of adult diabetic patients with a routine follow-up at the diabetic follow-up clinic of Debark hospital. Since there is no data on the level of knowledge and eyecheckup practice for diabetic retinopathy among diabetic patients in Ethiopia *p* = 50% was taken and margin of error was 5%. The generated sample size was found to be *n* = 384. After correcting for a finite population the sample size was 216. Considering 10% for non-response rate the total sample size was 238.All adults with diabetes mellitus aged ≥18 years, attending at Debark hospital diabetic follow-up clinic were included in the study. By using systematic random sampling, and taking their card number order every other participant was selected to be a sample.

### Operational definitions

#### Knowledge

Respondents who scored greater than or equal to the mean (≥5.55) of knowledge questions were considered to have good knowledge and those who scored below the mean were considered as having poor knowledge [5, 17].

#### Scoring

Participant’s knowledge of DR was assessed by 12 questions with a maximum score of 14 points. A numerical value of 1 for correct response and 0 for incorrect response was given.

#### Eye check-up practice

Participants who had ocular examination within the last 1 year were considered as having good eye checkup practice while those without any eye examination within the last 1 year were labeled as having poor eye checkup practice [9, 18].

#### Regular diabetic checkup

Participants who are undertaking investigations at least every 1 month since diagnosis of diabetes were considered as having regular diabetic checkup [5, 19].

#### Eye disease history

A participant who had experienced any type of eye diseases in the past 1 year.

### Data collection tool and procedures

A structured face to face interviewer administered questionnaire was used to collect the data. The questionnaire was developed from Indian guideline for conducting a knowledge, attitude and practice study for diabetic retinopathy [20]. The questionnaire from the Indian guideline was pretested on 5% of the sample size outside the study area at Debre-Tabour general Hospital and was little modified to make it suitable to the study population. The validity of the questionnaire was assessed by cronbach’s alpha. The cronbach’s alpha result was 0.8. Data regarding knowledge about DR, eye check-up practices for DR, socio-demographic variables such as age, sex, income, marital status, education, religion, occupation, ethnicity, health profile variables such as type of DM, duration of diabetics and history of eye disease were collected. Questions in the knowledge section included definition, risk factors, screening for diabetic retinopathy and treatment options for diabetic retinopathy. Eye check-up practice was assessed by eye check-up behavior, referral and regular diabetic follow-up of the adult diabetic patients with regard to diabetic retinopathy.

Two trained optometrists and two ophthalmic nurses from Debark hospital staffs were involved in the data collection. One optometrist supervised the data collection procedure. The participants were informed that participation in the study is voluntary and the information gathered will be used for academic, policy making and other societal problem solving purposes. Patients who were willing to participate in the study gave a verbal consent.

### Statistical analysis

After cleaning and coding, the collected data were entered to EPI info 7 and exported to Statistical Package for the Social Sciences (SPSS) version 20 for analysis. Both descriptive and analytical methods were used for analysis. Summary statistics, frequencies and cross tabulations were performed for the descriptive analysis of the data.

Binary logistic regression analysis was done to see the effect of independent variables on the dependent variables. Predictors with a *P*-value of ≤0.2 in the bivariable binary logistic regression were entered to the multivariable logistic regression analysis model by using enter method. The Hosmer-Lemshow goodness of fit statistic was used to assess whether the necessary assumptions for the application of multiple logistic regression were fulfilled. Multi co-linearity and reliability were checked using variation inflation factor and cronbach’s alpha respectively. The cronbach′s alpha scale was 0.8. Adjusted odds ratio with 95% confidence interval was used to determine the strength of the association and *P*-value less than 0.05 was considered as statistically significant.

## Results

### Socio-demographic characteristics of study participants

Two hundred thirty patients were participated in the study, giving a 96.68% response rate. The mean age of the participants was 49(±17.6) years. The median monthly income was 850 ETB [IQR of 500-3500ETB]. (Table 1).

**Table 1:** Socio-demographic characteristics of the study participants at Debark hospital, Northwest Ethiopia, 2018 (*n* = 230)

Variable	Frequency	Percentage
Sex		
Male	119	51.7
Female	111	48.3
Age (**year**)		
18–35	58	25.2
36–50	63	27.4
51–62	53	23.1
≥ 63	56	24.3
Residence		
Rural	123	53.5
Urban	107	46.5
Religion		
Orthodox	170	73.9
Muslim	60	26.1
Ethnicity		
Amhara	213	92.6
Tigrie	17	7.4
Marital status		
Single	51	22.2
Married	143	62.2
Divorced	12	5.2
Widowed	24	10.4
Occupation		
Farmer	77	33.5
Private	54	23.5
Government employed	47	20.4
Housewife	32	13.9
Retired	20	8.7
Education		
Can’t read and write	89	38.7
Can read and write	45	19.6
Elementary (1–8)	27	11.7
Secondary (9–12)	23	10.0
College and above	46	20.0
Monthly income (in ETB)		
≤ 500	88	38.3
501–850	23	10.0
851–3500	59	25.6
3501–8000	60	26.1

*n* = *s*ample size.

### Health profile of study participants

The median duration of diabetes mellitus from the time of diagnosis was 5 years [IQR 3–12]. Eighty-three (36.1%) of study subjects had a history of previous eye disease (Table 2).

**Table 2:** Health profile of study participants at Debark hospital, Northwest Ethiopia 2018 (*n* = 230)

Variable	Frequency	Percentage
Type of DM		
Type one	36	15.7
Type two	89	38.7
Don’t know	105	45.6
DM duration (years)		
≤ 2	64	27.8
3–5	55	23.9
6–12	64	27.8
13–25	47	20.5
Hypertension		
Yes	57	24.8
No	168	73.0
Don’t know	5	2.2
History of eye disease		
Yes	83	36.1
No	147	63.9

### Knowledge about diabetic retinopathy among study participants

The mean (+SD) knowledge score of study participants about DR was 5.55 (±4.66) with a maximum possible score of 14. One hundred twenty one [121 (52.6%)] participants had poor knowledge regarding diabetic retinopathy. Among those participants who had good knowledge about DR 25.6% were males and 33.9% were urban residents. One hundred sixty-seven (72.6%) of participants knew that diabetes affects the eye.

The respective correct response on risk factors for DR for poorly controlled blood glucose level, long duration of DM, hypertension, high body mass index, smoking and pregnancy were 42.6, 36.6, 26.1, 21.3, 12.2, 2.2% respectively. One hundred nine (66.5%) participants knew the importance of regular eye checkup. Only 14 (9.3%) mentioned laser as a treatment option for DR (Table 3).

**Table 3:** Knowledge of diabetic retinopathy among diabetic patients at Debark hospital, Northwest Ethiopia, 2018

Variable	Frequency	Percentage
Does diabetes affect the eye? (*n* = 230)		
Yes	167	72.6
No	63	27.4
Does diabetes cause blindness?		
Yes	151	67.4
No	73	32.6
What Eye condition does diabetes cause? (*n* = 164)		
Diabetic retinopathy	44	26.8
Cataract	31	18.9
Glaucoma	11	6.7
Don’t know	78	47.6
What are the risk factors for developing diabetic eye disease? (*n* = 164)		
Poorly controlled blood sugar	98	59.8
Duration of diabetes	23	14
Hypertension	64	39
High BMI	12	7.32
Pregnancy	49	29.9
Smoking	77	47
I don’t know	23	14.02
Should a person with diabetes cheek his/her blood pressure?		
Yes	132	80.5
No	8	4.9
I don’t know	24	14.6
Is blood sugar control important in preventing blindness from diabetic Retinopathy?		
Yes	101	61.6
No	21	12.8
I don’t know	42	25.6
What is diabetic retinopathy? (*n* = 164)		
It is the same as cataract	6	3.7
It is high sugars in the eye	16	9.8
Changes in the blood vessels of the retina	30	18.2
It is high pressure in the eye	3	1.8
Don’t know	109	66.5
Should a person with DM need eye screening? (*n* = 164)		
Yes	117	71.3
No	2	1.3
Don’t know	45	27.4
How soon after the diagnosis need eye screening?		
Immediately	56	51.4
One year	21	19.2
Five years	32	29.4
Does a patient with diabetes mellitus needs a regular eye checkup		
Yes	48	29.3
No	83	50.6
I don’t know	33	20.1
Is diabetic eye disease treatable?		
Yes	52	31.7
No	78	47.6
Don’t know	34	20.7
What treatment options are available for DR?		
Medical	28	18.5
Laser	14	9.3
Surgery	20	13.3
Don’t know	88	58.7

### Source of information about diabetic retinopathy

**Fig. 1:**
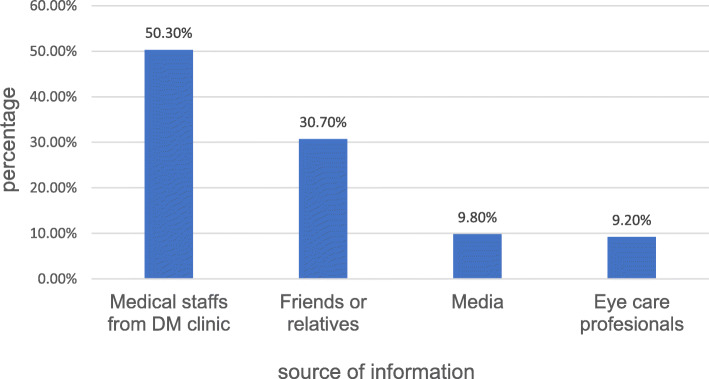
Source of information about diabetic retinopathy among diabetes patients at Debark hospital, Northwest Ethiopia, 2018 (*n* = 167)

The major sources of information about diabetic retinopathy for the respondents were medical staffs from diabetic clinic and followed by their relatives or friends (Fig. 1).

### Eye check-up practice of diabetic retinopathy

One hundred thirty-nine [139 (60.4%)] had poor eye check-up practice regarding diabetic retinopathy. Among those who examined their eyes 65.6% reported that they got dilated fundus examination (Table 4).

**Table 4:** Eye check-up practice of diabetic patients at Debark hospital, Northwest Ethiopia, 2018 (*n* = 230)

Variable	Frequency	Percentage
Have you ever been referred to check your eyes?		
Yes	97	42.2
No	133	57.8
Have your eyes been examined?		
Yes	95	41.5
No	134	58.5
How many times have your eyes been examined in the last one year? (*n* = 95)		
None	4	4.2
Once	56	58.9
Twice	24	25.3
3 times	11	11.6
How often did you check your blood sugar level?		
Monthly	177	77.0
Every two months	40	17.3
Every three months	13	5.7

The reasons for not getting eye checkup were reported as lack of visual symptoms (didn’t think it necessary) in 56.7% of participants and followed closely by 32.1% who felt they needed to be referred by their physician before getting their eyes examined and lack of convenient facility in 11.2% of participants.

### Factors associated with knowledge of diabetic retinopathy

Age, religion, residence, ethnicity, occupation, education, monthly income, type of DM, duration of DM from diagnosis and previous eye disease were significant from the bivariable analysis and entered in to multivariable analysis. The result of the multivariable binary logistic regression analysis showed that, the odds of good knowledge among participants from urban was 2.6 times (AOR =2.65, CI: 1.16–6.07) more as compared to those from rural area. Similarly, the odds of good knowledge among participants who had monthly income of 3501–8000 ETB was 4.5 times (AOR = 4.54, CI: 1.31–15.7) more as compared to those who had ≤500 ETB monthly income. The odds of good knowledge among participants having a diabetes duration of [6–12] years was 4.4 times (AOR = 4.42, CI: 1.44–13.5) more as compared to participants having a DM duration of ≤2 years. The odds of good knowledge among participants who had previous eye disease was 5.5 times (AOR = 5.5, CI: 2.3–13.0) more as compared to those who hadn’t it (Table 5).

**Table 5:** Factors associated with knowledge of diabetic retinopathy at Debark hospital, Northwest Ethiopia, 2018 (*n* = 230)

Variable	Knowledge		COR (95% CI)	AOR (95% CI)
	Good	Poor		
Sex				
Female	50	61	1.00	
Male	59	60	1.2 (0.71–2.01)	
Age (in years)				
18–35	18	40	1.00	1.00
36–50	33	30	2.44 (1.16–5.14)	3.16 (0.6–16.4)
51–62	26	27	2.14 (0.98–4.64)	2.58 (0.49–13.6)
≥ 63	32	24	2.96 (1.37–6.38)	3.17 (0.5–20.0)
Residence				
Rural	31	92	1.00	1.00
Urban	78	29	7.98 (4.42–14.3)	2.65 (1.16–6.07)*
Religion				
Muslim	35	25	1.00	1.00
Orthodox	74	96	0.55 (0.3–0.99)	0.79 (0.32–2.0)
Ethnicity				
Tigre	16	11	1.00	1.00
Amhara	93	110	0.58 (0.25–1.31)	0.56 (0.13–2.23)
Marital status				
Single	22	29	1.00	
Married	68	75	1.19 (0.62–2.27)	
Others	19	17	1.47 (0.62–3.47)	
Education				
Can’t read & write	26	63	1.00	1.00
Can read & write	16	29	1.3 (0.6–2.8)	0.8 (0.27–2.41)
Elementary (1–8)	15	12	3.0 (1.2–7.3)	1.3 (0.34–4.86)
Secondary (9–12)	14	9	3.7 (1.4–9.7)	1.52 (0.40–5.82)
College and above	38	8	11.5 (4.7–27.9)	1.62 (0.36–7.23)
Occupation				
Farmer	17	60	1.00	1.00
Private	30	24	4.4 (2.06–9.43)	3.15 (0.93–10.6)
Government	36	11	11.5 (4.8–27.3)	2.36 (0.54–10.2)
Housewife	13	19	2.4 (0.9–5.8)	0.81 (0.25–2.66)
Retired	13	7	6.5 (2.2–19.0)	1.3 (0.25–6.84)
Monthly income (in ETB)				
≤ 500	18	70	1.00	1.00
501–850	10	13	2.99 (1.13–7.92)	1.86 (0.52–6.73)
851–3500	32	27	4.6 (2.22–9.55)	1.61 (0.56–4.58)
3501–8000	49	11	17.3 (7.52–39.8)	4.54 (1.31–15.7)*
Type of DM				
Type 1	16	20	1.00	1.00
Type 2	65	24	3.38 (1.51–7.59)	2.76 (0.66–11.5)
Don’t know	28	77	0.45 (0.21–0.99)	0.71 (0.18–2.67)
Duration of DM (in years)				
≤ 2	17	47	1.00	1.00
3–5	18	37	1.34 (0.6–2.9)	2.16 (0.7–6.63)
6–12	40	24	4.6 (2.2–9.7)	4.4 (1.4–13.5) **
13–25	34	13	7.2 (3.1–16.8)	2.6 (0.7–9.7)
Previous eye disease				
Yes	63	20	6.9 (3.7–12.7)	5.5 (2.3–13.0)***
No	46	101	1.00	1.00

*p < 0.05, **p < 0.01, ***p < 0.001, Others = Widowed & Divorced

### Factors associated with eye check-up practice of diabetic retinopathy

Age, residence, marital status, occupation, educational level, knowledge of type of DM, monthly income, duration of DM, previous eye disease and knowledge of DR were significant on bivariable binary logistic regression model. In the multivariable binary logistic regression analysis duration of DM, previous eye disease and knowledge of DR were significantly associated with good eye checkup practice. The odds of good eye check-up practice among participants with a diabetes duration of [13–25] years was nearly 4 times (AOR = 3.77, CI: 1.05–13.5) more as compared to participants with a DM duration of ≤2 years. The odds of good eye check-up practice among participants having previous eye disease was 2.4 times (AOR = 2.47, CI: 1.09–5.62) more as compared to those who hadn’t it. The odds of good eye checkup practice among participants who had good knowledge of DR was 17 times (AOR = 17.5, CI: 5.97–51.3) more as compared to those who had poor knowledge (Table 6).

**Table 6:** Factors associated with eye checkup practice of DR at Debark hospital, Northwest Ethiopia, 2018

Variable	Eye checkup practice		COR (95% CI)	AOR (95% CI)
	Good	Poor		
Sex				
Female	44	67	1.00	
Male	47	72	0.99 (0.58–1.68)	
Age (in years)				
18–35	10	48	1.00	1.00
36–50	31	32	4.65 (2.0–10.7)	4.04 (0.99–14.9)
51–62	21	32	3.15 (1.31–7.56)	1.49 (0.31–7.09)
≥ 63	29	27	5.15 (2.18–12.1)	1.4 (0.27–7.3)
Residence				
Rural	34	89	1.00	1.00
Urban	57	50	2.98 (1.72–5.16)	1.01 (0.39–2.25)
Religion				
Muslim	26	34	1.00	
Orthodox	65	105	0.81 (0.44–1.47)	
Ethnicity				
Tigre	13	14	1.00	
Amhara	78	125	0.67 (0.25–1.5)	
Marital status				
Single	14	37	1.00	1.00
Married	56	87	1.7 (0.84–3.43)	0.63 (0.18–2.25)
Others	21	15	3.7 (1.45–9.08)	1.85 (0.38–9.08)
Occupation				
Farmer	22	55	1.00	1.00
Private	19	35	1.35 (0.64–2.86)	0.46 (0.12–1.76)
Government	23	24	2.39 (1.12–5.1)	0.41 (0.09–1.92)
Housewife	14	18	1.94 (0.82–4.57)	0.90 (0.25–3.19)
Retired	13	7	4.64 (1.63–13.2)	1.15 (0.2–6.63)
Education				
Can’t read & write	30	59	1.00	1.00
Can read &write	12	33	0.71 (0.32–3.27)	0.36 (0.11–1.23)
Elementary (1–8)	11	16	1.35 (0.56–3.27)	0.62 (0.16–2.45)
Secondary (9–12)	12	11	2.14 (0.85–5.43)	1.42 (0.36–5.66)
College and above	26	20	2.55 (1.23–5.3)	0.55 (0.13–2.38)
Monthly income (in ETB)				
≤ 500	19	69	1.00	1.00
501–850	13	10	4.72 (1.79–12.4)	3.85 (0.99–14.9)
851–3500	19	40	1.72 (0.81–3.63)	0.61 (0.19–1.93)
3501–8000	40	20	7.26 (3.46–5.36)	1.76 (0.49–6.33)
Type of DM				
Type 1	10	26	1.00	1.00
Type 2	50	39	3.33 (1.44–7.73)	1.2 (0.24–5.93)
Don’t know	31	74	1.09 (0.47–2.53)	1.04 (0.21–5.13)
Duration of DM (in years)				
≤ 2	10	54	1.00	1.00
3–5	17	38	2.41 (1.0–5.85)	2.36 (0.73–7.68)
6–12	33	31	5.74 (2.49–13.2)	2.9 (0.96–8.77)
13–25	31	16	10.4 (4.2–25.8)	3.77 (1.05–13.5)*
Previous eye disease				
Yes	54	29	5.53 (3.08–9.93)	2.47 (1.09–5.62)*
No	37	110	1.00	1.00
Knowledge of DR				
Good	75	34	14.4 (7.4–28.1)	17.5 (5.97–31.3) ***
Poor	16	105	1.00	1.00

*p < 0.05, **p < 0.01, ***p < 0.001, Others = Widowed & Divorced

## Discussion

In this study 47.4% [95% CI: 41.7–53.9%] of study participants had good knowledge regarding diabetic retinopathy. This result is in line with the study done in India [21] which was reported as 47%. However, the result in this study is lower than the studies conducted in Bangladesh [17] and Saudi Arabia [22] which were 76 and 64% respectively. This difference might be due to lack of organized diabetic education facilities, less participation of media and non-governmental organizations in awareness creation about diabetic retinopathy in present study setting.

One hundred sixty-seven (72.6%) [95% CI: 67.0–78.4%] study participants thought that diabetes affects the eye which is consistent with the study done in India [6], Saudi Arabia [12] and Nigeria [23] which were reported as 74.3, 75.6 and 69.9% respectively. This might be due to similarity in the study design and setting. In Contrary, this is lower than the study conducted in Malaysia [24], South Africa [25] and Kenya [26] which were reported as 87.2, 97.3 and 83% respectively. This difference might be due to the study participants in Malaysia and South Africa were patients from retina clinic who came for routine eye examination and they might have more exposure on DR information.

In this study 44 (26.8%) participants were able to mention diabetic retinopathy as a complication of DM and 30 participants (18.3%) defined diabetic retinopathy correctly. This result is lower than the study reported in Bangladesh [17] which was 55%. It might be due to limited source of information and inadequate involvement of the media in the present study.

Knowledge on risk factors like poor control of blood glucose level was 42.6% and longer duration of DM was 36.6%, this finding is higher as compared to a study done in India [6] which were reported as 33.7 and 17.9% respectively. This could be due to variation in the level of information given by the physicians on risk factors and their consequences.

In this study one hundred-nine (66.6%) study participants realized the importance of regular eye check-up. This finding is consistent with a study done in Nigeria [13] which was 66.9%. Knowledge on treatment option of DR was very low, only 14(9.3%) participants knew laser as a treatment option for DR. This result is comparable to a similar study done in India [6] where 4.7% of participants mentioned laser as treatment option. This might be due to knowledge on treatment of DR most likely acquired from eye clinic environment rather than DM follow up clinic.

This study also showed that 39.6% [34.3–45.7%] of participants had good eye checkup practice regarding diabetic retinopathy and 60.4% of participants had poor eye checkup practice regarding DR. This finding is in line with the study done in South Africa [25] and Bangladesh [17] where the practice of eye examination was reported as 37% in both studies. On the other hand, the result from the present study is lower than the result obtained from Australia [27] [71%], Malaysia [24] [50%], Ghana [9] [65.4%], Kenya [26] [50%]. This could be due to variation in socio-demographic characteristics and low access to health care in the present study. In contrast, the result from this finding is higher than the result reported from India [21] [21.6%] and Pakistan [25.6%]. This could be explained by the variation in study design and target population where the study done in Pakistan was a comparative population-based study.

The reasons for not getting eye examination were lack of visual symptoms in 56.7% participants and followed by 32.1% who felt they needed to be referred by their physician before getting their eyes examined. This result is consistent with the finding reported from Nigeria [23]. This is explained by poor perception of participants about the need for regular eye examination.

According to this study the odds of good knowledge among urban residence participants was 2.6 times more as compared to those from rural residence. This is supported by a study conducted in Bangladesh [28]. This could be explained by people living in urban have multiple source of information to know about diabetic retinopathy; health centers, mass media and higher people interactions than those live in rural.

Higher monthly income level was significantly associated with good knowledge in the present study. This finding is consistent with studies done in India [11, 15], where good knowledge was positively associated with higher socioeconomic status. The possible explanation for this finding could be people with high income level might have more exposure to Medias and inter personal interaction than those who had low income level.

It is also indicated that study participants who knew the type of DM diagnosed were more knowledgeable regarding diabetic retinopathy than those who didn’t know.

This study had revealed a positively association between long duration of DM and good knowledge of diabetic retinopathy. This finding is supported by studies conducted in Nigeria and Iran [13, 14]. This could be due to frequent contact of the participants with the health care provider which creates opportunity to get information regarding diabetes complications.

History of eye disease at least once in their life was significantly associated with good knowledge about DR in this study. This might be due to the health education given for patients coming for eye examination help them to acquire some basic knowledge about the disease.

In the previous studies, sex [9, 12] and age [12, 14] were significantly associated with knowledge of DR. However, in the present study sex and age were not significantly associated with knowledge of DR. In this study educational level didn’t significantly associated with knowledge. In contrast, educational level was significantly associated with knowledge of DR in a studies done in Nigeria and India [11, 13]. This might be due to variation in study participants’ characteristics, where most of the study participants were illiterate in present study and this could mask the significance of educational level in present study.

Longer duration of DM was positively associated with good eye checkup practice in the present study. This is supported by a study conducted in Bangladesh [28] and India [29]. This might be due to as the disease duration increases their knowledge to eye screening increases as evidenced by the association of knowledge and disease duration. This might be also due to continued counseling and health education programs.

Good knowledge of diabetic retinopathy was significantly associated with good eye check-up practice. This result is supported by the studies conducted in Ireland [18], Bangladesh [17] and Ghana [9]. This might be due to having a good knowledge creates a firm belief in need for annual eye check-up and as showed the major reason for poor eye check-up was the wrong perception of eye screening needed only if there is a visual symptom.

The odds of good eye check-up practice among participants who had history of previous eye disease was 2.4 times more as compared those who hadn’t it. This finding is supported by a study conducted in Ireland [18]. This might be due the fact that most patients go for eye examination if they develop a visual symptom.

In previous studies age [10, 11] and ethnicity [16] were significantly associated with eye check-up practice. However, the data in the current study didn’t support this. This variation might be due to difference in study design, because the study done in United Kingdom used qualitative study design and also it might be due to small number of cases in the present study.

Over all, even though this research provides information regarding knowledge, eye checkup practice and associated factors of diabetic retinopathy among adult diabetic patients attending diabetic follow-up clinic at Debark hospital in Northwest Ethiopia, it has the following limitations. Some questions like history of eye disease may be forgotten by the participant so that it might be affected by recall bias. More over eye checkup practice may be affected by attitude of patient, which this research doesn’t address the attitude of the participants towards eye check up practice.

From the findings of this study we recommend the national, regional, zonal and Debark Hospital health authorities to set a guideline that includes advice for diabetic patients about diabetic retinopathy as one component of the standard treatment guideline. More over it is better if the national regional and zonal mass media and the hospital coordinator set a regular time to deliver education about diabetic retinopathy and other diabetic complications with the aim of improving the knowledge and eye checkup practice of patients with diabetes mellitus. Other factors that affect diabetic retinopathy including blood pressure control, glycemic control and lipid control shall be the components of patient education. We also recommend future researchers to conduct further studies on knowledge about diabetic retinopathy and eye check-up practice among adult diabetic patients including the participant’s attitude and other health aliments in a multicenter setting with a larger size population.

## Conclusions

In conclusion, finding from this study revealed a good knowledge on nearly half (47.4%) of study participants and good eye check-up practice on more than one third (39.6%) of study participants regarding diabetic retinopathy among diabetes mellitus patients attending debark hospital, Northwest Ethiopia. Being urban resident, higher monthly income, knowledge of type of DM diagnosed, history of eye disease and longer duration of diabetes were the factors positively associated with good knowledge of participants. Similarly, factors associated with good eye checkup practice were longer duration of diabetes mellitus, previous eye disease and good knowledge of diabetic retinopathy.

## Supplementary Information

**Additional file 1.**

## Data Availability

The datasets used and/or analyzed during the current study are available from the corresponding author on reasonable request.

## Abbreviations

AOR: Adjusted Odds Ratio
BMI: Body Mass Index
CI: Confidence Interval
DM: Diabetes Mellitus
DR: Diabetic Retinopathy
ETB: Ethiopian Birr
EPI INFO: Epidemiological Information
IQR: Inter quartile Range
SD: Standard Deviation
SPSS: Statistical Package for Social Sciences
