# Supplementary material for: Knowledge about diabetic retinopathy, eye check-up practice and associated factors among adult patients with diabetes mellitus attending at debark hospital, Northwest Ethiopia
Source: BMC Ophthalmol. 2020 Nov 18;20:453. doi: 10.1186/s12886-020-01730-4 (PMC7672967; doi:10.1186/s12886-020-01730-4)
Supplement: Supplementary file 1 — Additional file 1. [file 12886_2020_1730_MOESM1_ESM.docx]

Annex 4: Questionnaire Format (English Version)

A structured questionnaire form for knowledge, eye check-up practice and associated factors of diabetic retinopathy among adult diabetic patients in Debark hospital, Northwest Ethiopia.

**Introduction**

Good morning/afternoon, my name is --------------------------------. I am a member of a research group which studies the knowledge, eye check-up practice and associated factors of diabetic retinopathy among adult diabetic patients in Debark hospital. You are selected and kindly invited to be one of the study participants in our project. Your truth full answers for all of our questions are very important to determine the knowledge, eye check-up practice and associated factors of diabetic retinopathy among diabetic patients. Your answers will be confidential and secret. It is your right not to participate in the study, stop at any time or skip any question you do not want to answer. We will take a maximum of 20 minutes to complete the questionnaire.

Thank you.

Next, I will read a consent, which assures your interest to participate.

Do I have your permission to continue?

If yes thank you and continue --------------

If no, thank you and go to next study subject --------------

Data collector

Name ----------------------- signature ------------------------ date -------------------

Checked by supervisor

Name --------------------- signature---------------------- date------------------

**Part 1. Socio-demographic and health related data**

| Serial No, | Question | Response |
| --- | --- | --- |
|  | Code number |  |
| 1 | Sex | 1.Male  2.Female |
| 2 | Age in year |  |
| 3 | Religion | 1. Orthodox 3. Protestant  2. Musilim 4.Other……………. |
| 4 | Residence | 1.Urban  2. Rural |
| 5 | Marital status | 1. Single (Never married) 3. Divorced  2. Married 4. Widowed |
| 6 | Educational level | 1.Can’t read and write  2. Can read and write with no formal education  3. Primary (1 -8)  4. Secondary  5. Tertiary (College/University) |
| 7 | Occupation | 1.Farmer  2.Daily laborer  3. Goverment employed  4. House wife  5. Retired  6) Merchant  7) Others specify……………… |
| 8 | Ethnicity | 1.Amhara  2.Tigrie  3. Kemant    4. Oromo 5. Other……………... |
| 9 | Family monthly income (in Ethiopian birr) |  |
| 10 | How long have you been diabetic? |  |
| 11 | Type of DM | 1.Type-1  2.Type-2  3. Don’t know |
| 12 | Do you have Hypertension? | 1.yes  2.No  3. Don’t know |
| 13 | Previous history of eye disease in the past one year? | 1.yes  2.No  3. Don’t know |

Part 2 Knowledge of Diabetic retinopathy

| Serial No, | Question | Response |  |  |
| --- | --- | --- | --- | --- |
| 14 | Does diabetes affect the eye? | 1.Yes  2.No  3.Don’t know |  |  |
| 15 | Can diabetes cause blindness? (If answered “No” to both the above questions #14 and #15, please go to question #27) | 1.Yes  2.No  3.Don’t know |  |  |
| 16 | What eye condition does diabetes specifically cause in the eyes? | 1.Diabetic retinopathy  2.Cataract  3. Glaucoma  4. Don’t know  5. Others………………… |  |  |
| 17 | What is diabetic retinopathy? | 1. It is the same as cataract.  2. It is high sugars in the eye.  3.It is changes in the blood vessels of the retina due to diabetes  4.It is high pressure in the eye  5.Don’t know |  |  |
| 18 | What are the risk factors for developing diabetic eye disease? (You can choose more than one answer) | 1.Poorly controlled blood sugar  2.Duration of diabetes  3.Hypertension  4.High BMI  5.Pregnancy  6.Smoking  7.I don’t know |  |  |
| 19 | Should a person with diabetes cheek his/her blood pressure? | 1.yes  2.No  3.don’t know |  |  |
| 20 | Is blood sugar control important in preventing blindness from diabetic Retinopathy? | 1.Yes  2.No  3.Don’t know |  |  |
| 21 | Is diabetic eye disease treatable? (If answered “No” to the above question, please go to question #23) | 1.Yes  2.No  3.Don’t know |  |  |
| 22 | What are the treatment options available for diabetic eye disease?  (You can choose more than one answer) | 1.Medical (Injections inside the eyes)  2.Laser burns inside the eyes  3.Surgery  4.don’t know |  |  |
| 23 | Should a person with diabetes mellitus need eye screening? | 1.Yes  2.No  3.Don’t know (If answered “No”  to this question, please go to question #25) | |  |
| 24 | How soon after the diagnosis has been made should that person visit the specialist eye doctor? | 1.Immediately after diagnosis of DM  2.One year after diagnosis  3.Five years after diagnosis  4.Other _________________ | |  |
| 25 | Does a diabetic patient need a regular eye checkup? | 1.Yes  2.No  3.I don’t know | |  |
| 26 | How did you come to know about DM affecting the eyes? | 1.Health professional at the diabetes follow up clinic  2.eye care professional’s  3.Family member/relative/friend with diabetes  4. TV, magazines, other media 5.Other………….. | |  |

Part 3. Eye check-up practice of diabetic retinopathy

|  |  |  |
| --- | --- | --- |
| 27 | Have you ever been referred to see an eye doctor? | 1.Yes  2.No  3.Don’t know |
| 28 | Have your eyes been examined by an eye doctor after the diagnosis of Diabetes? | 1.Yes  2.No  3.Don’t know (If answered “No” to the above question, please go to question #31) |
| 29 | If “Yes” how many times in the last one year? | 1. None  2. Once  3.Twice  4.Three times or more |
| 30 | What kind of eye exam do you get? | 1. Vision 2. Checking for eye glass 3. Dilated examination of the back of the eye 4. Slit lamp/torch examination 5. Other……………………… |
| 31 | If “No” to question #28, why? | 1.I did not think it was necessary  2.I was not advised to by my doctor  3.Financial restrictions  4.Lack of convenient facility  5.Others (Specify) |
| 32 | Do you check your blood sugar? | 1. Yes  2. No  3.I don’t know  If yes, how often? _____________ |
